# Phase-Dislocation-Mediated High-Dimensional Fractional Acoustic-Vortex Communication

**DOI:** 10.34133/research.0280

**Published:** 2023-12-01

**Authors:** Ruijie Cao, Gepu Guo, Wei Yue, Yang Huang, Xinpeng Li, Chengzhi Kai, Yuzhi Li, Juan Tu, Dong Zhang, Peng Xi, Qingyu Ma

**Affiliations:** ^1^ School of Computer and Electronic Information, Nanjing Normal University, Nanjing 210023, China.; ^2^ Department of Biomedical Engineering, College of Future Technology, Peking University, Beijing 100871, China.; ^3^ Institute of Acoustics, Nanjing University, Nanjing 210093, China.; ^4^ National Biomedical Imaging Center, Peking University, Beijing 100871, China.

## Abstract

With unlimited topological modes in mathematics, the fractional orbital angular momentum (FOAM) demonstrates the potential to infinitely increase the channel capacity in acoustic-vortex (AV) communications. However, the accuracy and stability of FOAM recognition are still limited by the nonorthogonality and poor anti-interference of fractional AV beams. The popular machine learning, widely used in optics based on large datasets of images, does not work in acoustics because of the huge engineering of the 2-dimensional point-by-point measurement. Here, we report a strategy of phase-dislocation-mediated high-dimensional fractional AV communication based on pair-FOAM multiplexing, circular sparse sampling, and machine learning. The unique phase dislocation corresponding to the topological charge provides important physical guidance to recognize FOAMs and reduce sampling points from theory to practice. A straightforward convolutional neural network considering turbulence and misalignment is further constructed to achieve the stable and accurate communication without involving experimental data. We experimentally present that the 32-point dual-ring sampling can realize the 10-bit information transmission in a limited topological charge scope from ±0.6 to ±2.4 with the FOAM resolution of 0.2, which greatly reduce the divergence in AV communications. The infinitely expanded channel capacity is further verified by the improved FOAM resolution of 0.025. Compared with other milestone works, our strategy reaches 3-fold OAM utilization, 4-fold information level, and 5-fold OAM resolution. Because of the extra advantages of high dimension, high speed, and low divergence, this technology may shed light on the next-generation AV communication.

## Introduction

Manifested by helical wavefronts, the orbital angular momentum (OAM) of acoustic-vortex (AV) beams provides a new degree of freedom for information multiplexing in acoustic communications [1–5]. Although the channel capacity of AV beams can be boosted using more integer OAMs [6], the implementation of high-order topological charges (TCs) through an active transducer array or passive metamaterials struggles with the problems of complex structure and insufficient flexibility [7–9] with severe divergence caused by the large vortex radius [10–12] of high-order TCs. If the fractional OAMs (FOAMs) with a small enough interval in a limited TC scope are used, an infinitely expanded channel capacity can be realized by the possible unlimited OAM modes with an approximately identical vortex radius, and the huge potential to improve the channel capacity with reduce divergence [13–15] has been demonstrated. However, the orthogonality-based method of OAM decoding is no longer applicable to fractional vortex beams in theory [16]. Two methods of recognizing FOAMs have been developed in optics. One is to measure the displacement of the focal spot or count the number of interference fringes with an additional incident beam [17,18]. The other is based on image recognition via machine learning [19–21]. The FOAM resolution of 0.01 between 1.93 and 2.00 serving as 8-bit independent channels has been realized in optics [22].

Although these technologies in optics can provide effective references, the FOAM has not been implemented as an independent information carrier in AV communications, which may be limited by the restrictions below. First, different from optical imaging by a camera, the speed and efficiency of data transmission are severely restricted by the necessary acoustic field scanning [23,24]. Then, when the fractional AV (FAV) beam propagates through realistic circumstances, the channel capacity may be degraded by the strengthened cross-talk between nearby FOAMs caused by the field distortion in both pressure amplitude and phase [7]. Moreover, the FAV propagates helically around the beam axis with the phase distribution varying with the propagation distance, which brings a heavy burden to recognize the multiplexed FOAMs [25]. Last, the field difference between FAVs with adjacent FOAMs is too tiny to distinguish using the traditional scanning measurement approaches [22].

Here, on the basis of the combination of pair-FOAM (P-FOAM) multiplexing, circular sparse sampling, and convolutional neural network (CNN), a phase-dislocation-mediated high-dimensional FAV communication protocol in acoustics is proposed for the first time. The azimuthal phase dislocation formed by the interaction of P-FOAMs is demonstrated to be uniquely corresponding to the fractional TC and independent of the propagation distance, which provides important physical guidance to recognize FOAMs and reduce sampling points from theory to practice. On the basis of the properties, holographic signals obtained through the single/dual-ring sparse sampling instead of the 2-dimensional scanning measurement under the nonideal conditions are trained by a straightforward CNN without involving experimental datasets. In addition, the impact of the transmission distance and the noise level on the recognition accuracy are systematically investigated to analyze the robustness. The advantages of the FAV communication are proved by the high dimension (10-bit information transmission with 32-point dual-ring sampling) and high-resolution (0.025 with 128-point single-ring sampling) recognition of FOAMs. The outcomes verify the feasibility of infinitely expanding the channel capacity in a limited OAM scope.

## Results

### Phase-mediated recognition of FOAMs

**Fig. 1.**
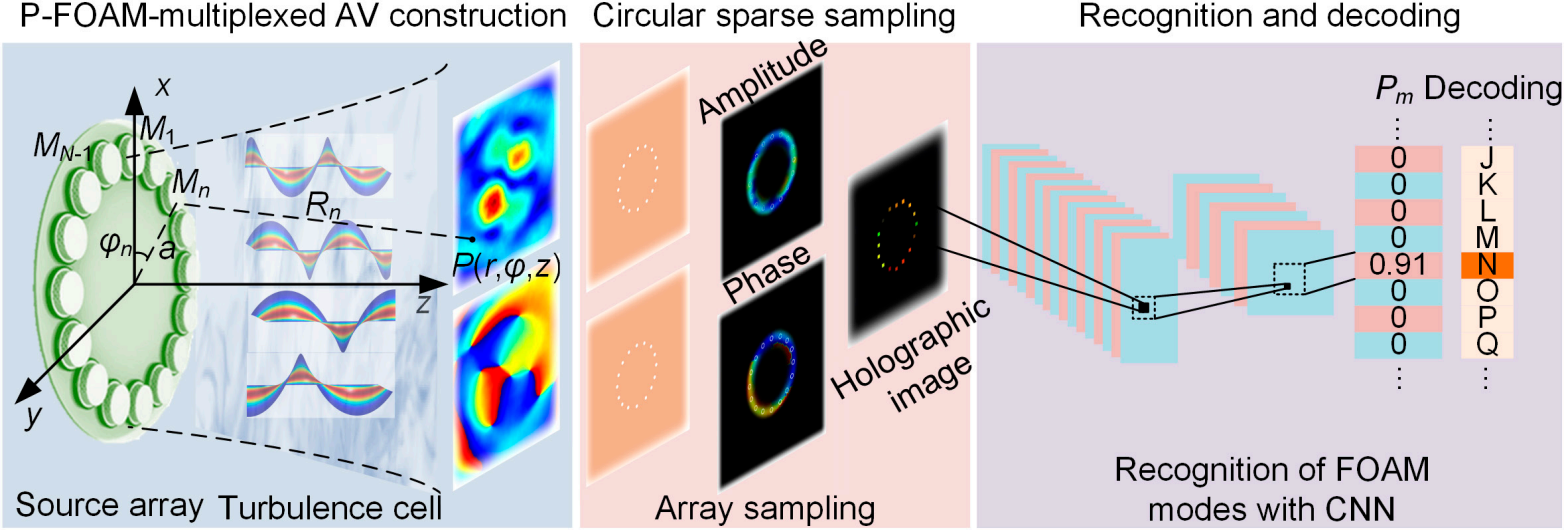
Principle of the phase-dislocation-mediated FAV communication based on circular sparse sampling and machine learning. Our approach can be divided into 3 main parts, including the P-FOAM-multiplexed beam construction, circular sparse sampling, and FOAM recognition and decoding. A single-ring source array is used to construct P-FOAMs, where a turbulence cell is used to approach the nonideal transmission condition. The acoustic pressures are acquired by the sparse sampling around the single/dual-ring, and holographic images including both pressure and phase are taken as the training input of CNN to classify P-FOAMs. The maximum classified probability of *P_m_* = 0.91 indicates the decoded result of transmitting the letter “N”.

The principle of the phase-dislocation-mediated FAV communication based on circular sparse sampling and machine learning is shown in Fig. 1, including the processes of field construction, data transmission, pressure detection, and OAM identification. The coupled FAV carrying a pair of opposite OAMs is designed to guide the decoding of P-FOAMs by the phase dislocation of pressure valleys. As is well known, the cross-sectional distribution of an AV of *m*th integer order is featured by the annular pressure distribution with a phase spiral around the vortex center. It can be expressed as *p*(*r*, *φ*, *z*_0_, *m*) = *A*(*r*, *m*) exp [*jγ*(*r*, *m*)] exp (*jmφ*) [26], where *φ* is the azimuth from 0 to 2*π* and *A*(*r*, *m*) and *γ*(*r*, *m*) represent the transmitter-determined distributions of pressure and phase, respectively. Note that the distinctive spiral distribution of FAVs can be described by the superposition of AVs of infinite integer orders as $\begin{array}{c} \text{exp}\left(\mathrm{jl\varphi}\right)=\frac{\text{exp}\left(\mathrm{j\pi l}\right)\text{sin}\left(\pi ,l\right)}{\pi }{\text{∑}}_{m=-\infty }^{+\infty }\frac{\text{exp}\left(\mathrm{jm\varphi}\right)}{l-m} \end{array}$ [16,25], where *l* (*l* > 0) and *m* represent fractional and integer orders of OAMs, respectively. Then, the cross-sectional pressure map of the coupled FAV carrying a pair of opposite FOAMs (±*l*) can be described as:

$$\begin{array}{c} p\left(r,\varphi ,z_{0},\pm l\right)=\\ \frac{\text{sin}\left(\pi ,l\right)}{\pi }\sum_{m=-\infty }^{\infty }\frac{2\text{exp}\left[j\gamma \left(m,r\right)\right]A\left(m,r\right)}{l-m}\text{cos}\left(\mathrm{m\varphi}+\pi l\right). \end{array}$$ (1)

In essence, the pressure distribution of the coupled FAV is determined by the superposition of integer-order AVs with the weighted coefficient of sin(*πl*)/[*π*(*l* − *m*)], and the azimuth of phase dislocation for P-FOAMs is mainly decided by the nearby integer-order OAMs with the accurate rotation of *lπ*/*m*. Hence, the strict correspondence between the azimuth and TC provides a solid physical foundation for the high-precision recognition of FOAMs (Note S1). To approach the nonideal transmission condition and improve the anti-interference for FOAM decoding [27,28], a random phase screen based on the power spectrum is introduced by considering the interference in realistic circumstances, and the P-FOAM-multiplexed FAV of accomplishing the high-dimensional data transmission can be expressed as [29]:

$$p_{S}\left(r,\varphi ,z_{0}\right)=e^{\mathrm{j\psi}\left(x,y\right)}\sum_{s=1}^{S}\left[p\left(r,\varphi ,z_{0},\pm l_{s}\right)\right]$$ (2)

where *S* is the pair number of P-FOAMs and *ψ*(*x*, *y*) = *F*^−1^{*C* × *σ*(*k_x_*, *k_y_*)} denotes the random phase screen that causes the field distortion with the inverse Fourier transform *F*^−1^{⋅} and the spectral power variance *σ*(*k_x_*, *k_y_*) (Note S1) [30,31].

### Phase-dislocation-guided sparse sampling

**Fig. 2.**
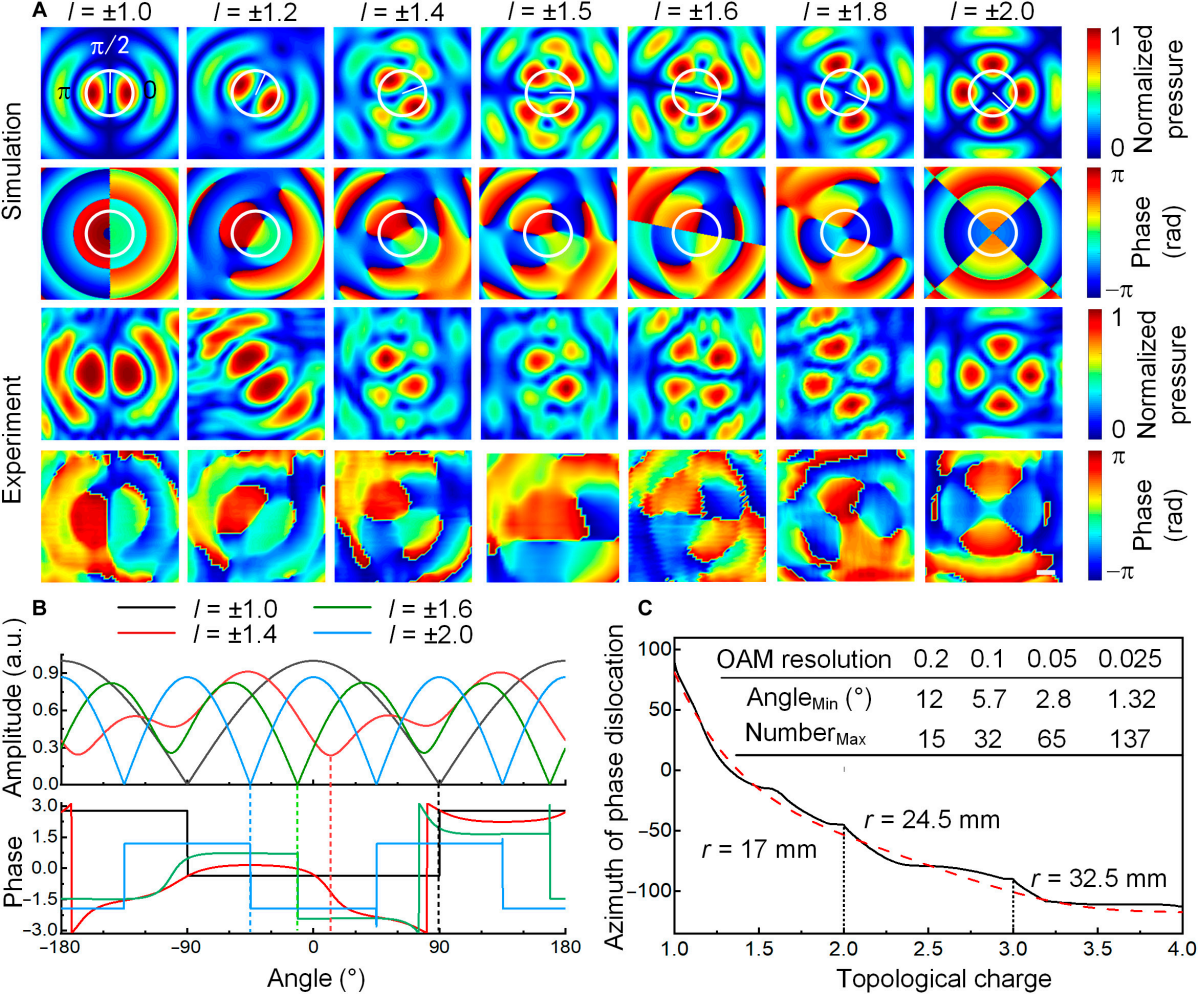
Feature analysis of coupled FAVs with *l* = ± 1.0 to ± 2.0. (A) Numerical and experimental cross-sectional distributions of pressure and phase for FAVs with P-FOAMs. The white ring indicates the circle of sparse sampling with *r* = 17 mm, which is the averaged radius of the pressure peaks for *l* = ±1 and ±2. (B) Sampled circular profiles of pressure and phase at *z*_0_ = 250 mm (29.41λ). (C) Simulated azimuthal distribution of the phase dislocation as a function of the TC with *l* = 1 to 4 at the OAM resolution of 0.01 (solid black line), with the fitted curve being presented as the red dashed line. The embedded table in (C) shows the relationship between the OAM resolution, the sampling number, and the minimum angle interval. Scale bar, 1 cm. a.u., arbitrary units.

In our experiment, the P-FOAM-multiplexed FAVs are constructed by the single-ring array of 16 sources through the phase-coded approach (Note S2). On the basis of numerical simulations and experimental measurements, cross-sectional distributions of pressure and phase at *z*_0_ = 29.41λ are illustrated in Fig. 2A. For *l* = ±1.0, the coupled FAV is split into 2 bilaterally symmetrical halves with the pressure valley (corresponding to the phase dislocation) along the vertical direction. Although the similar bilaterally symmetrical portions can be observed for P-FOAMs with the increased TCs of *l* = ±1.2, the low-pressure stripe rotates clockwise with the angle of 0.2*π* compared to that of *l* = ±1.0. The coupled FAV is mainly composed by AVs of nearby OAMs of *m* = ±1.0 with the azimuthal rotation of *lπ*/*m* = 1.2*π*, as described by Eq. 1. Considering the periodicity of |cos(*φ*)|, the rotation angle of 0.2*π* as noticed in simulations consists well with the theoretical angles of 0.2*π* and 1.2*π*. Especially for *l* = ±1.5, 2 new phase singularities emerge by the splitting of the phase singularity and, hence, result in 4 uneven petals. By further increasing *l*, the new phase singularities rotate in the clockwise direction and move toward the origin, eventually form 4 uniform petals with the low-pressure stripes positioned at ±*π*/4 for *l* = ±2.0. Meanwhile, the vortex radius grows from 13.5 to 20.5 mm by raising the TC from 1.0 to 2.0, which can serve as a helpful benchmark for the radius selection of circular sparse sampling. The detailed process and feature analysis for the coupled FAVs are shown in Note S3.

To coordinate the rotational feature of the coupled FAV carrying different P-FOAMs and precisely locate the phase dislocation as possible, the averaged radius of *r* = 17 mm for *l* = ±1 and ±2 is selected to conduct the circular sparse sampling. Figure 2B shows the circular profiles of pressure and phase of coupled FAVs with different P-FOAMs. It is clear that the pressure profile for *l* = ±1 satisfies the function of |cos(*φ*)|. The phase dislocations (corresponding to pressure valleys) emerge at the azimuths of *φ* =  ± 90^∘^. Taking *φ* = 90^∘^ as the reference, the azimuth of the phase dislocation moves to 10° and −11° for *l* = ±1.4 and ±1.6, respectively, and ultimately reaches −45° when *l* = ±2.0. Furthermore, the TC dependence of the azimuth of phase dislocation for *l* = ±1 to ±4 at the step of 0.01 in Fig. 2C shows a monotone decreasing tendency with a gradually reduced slope, which coincides well with the approximate inverse proportion of |*l*| ⋅ (*φ* + *π*) = 3π/2 and provides the fundamentals for the recognition of P-FOAMs. Note that, since the interaction of P-FOAMs with |*l*| < 0.5 cannot form a stable phase dislocation (Note S4 and Movie S1), the TC of |*l*| > 0.5 is chosen to realize FOAM recognition. As a result, the OAM resolution with respect to the sampling number and the corresponding minimum angle interval for the coupled FAVs with *l* = ±1 to ±2 are listed in the embedded table in Fig. 2C. Taking the FOAM resolution of 0.2 as an example, the minimum rotation angle of the phase dislocation is about Δ*ψ* = 12^∘^, and the required least sampling points of ⌈180^∘^/Δ*ψ*⌉ = 15 is achieved. The corresponding training curve and decoding results for nonmultiplexed single P-FOAM with the 16-point circular sampling are shown in Note S5. It shows a high accuracy of 99.54% with almost no cross-talk, which primarily demonstrates the stability of the phase-dislocation-guided data transmission.

### High-dimensional long-distance FOAM transmission

**Fig. 3.**
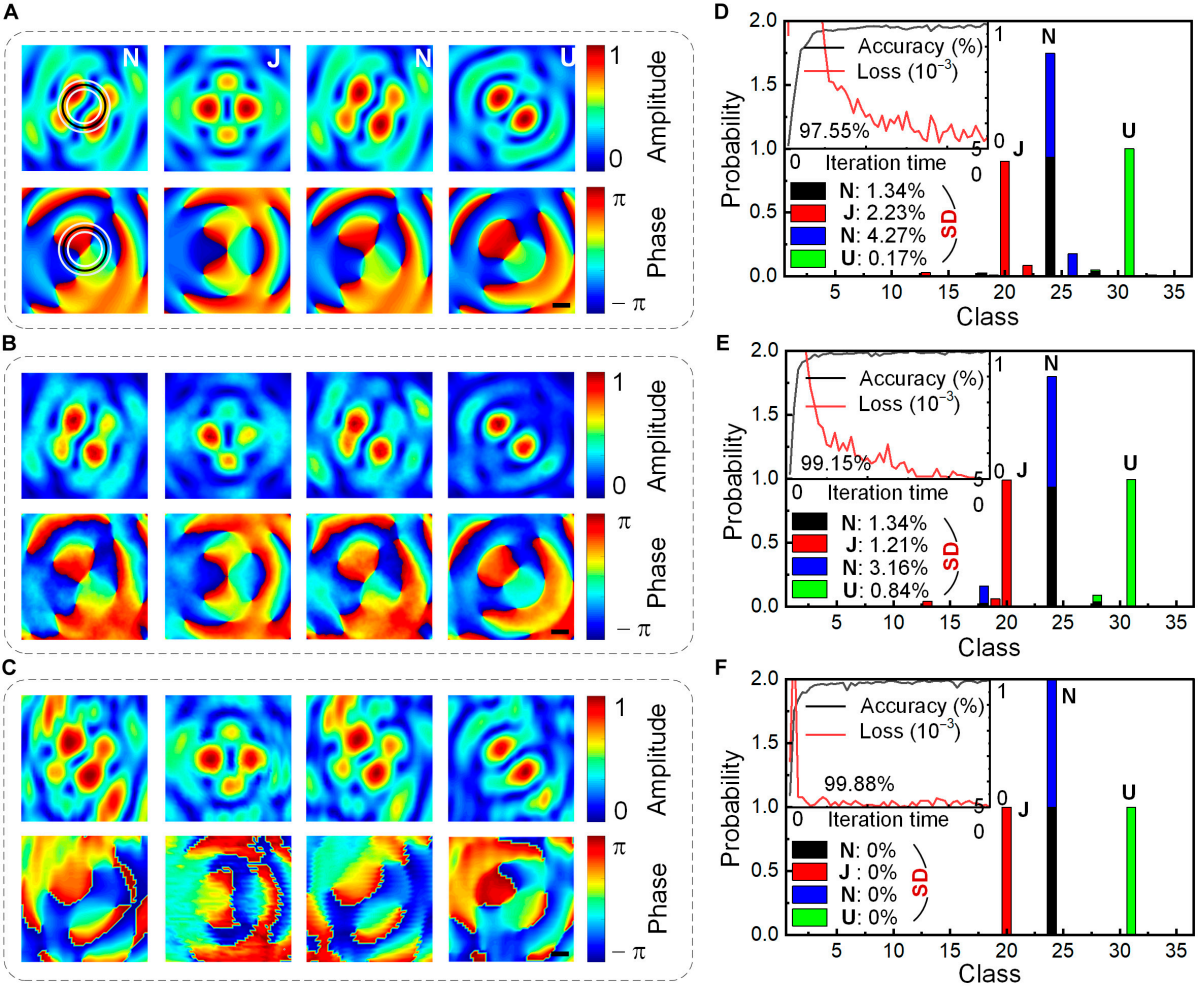
Recognition of P-FOAMs for transmitting letters “NJNU” using various circular sampling methods. Simulated cross-sectional distributions of pressure and phase at *z*_0_ = 29.41λ (A) without and (B) with turbulence and (C) the corresponding experimental results. Distributions of the probability (histogram), loss (red curve), and classification accuracy (black curve) with respect to the iteration time (top left), and the SDs for the sampling methods of (D) 16-point single-ring, (E) 32-point single-ring, and (F) 32-point dual-ring. Scale bars, 1 cm.

To further demonstrate the capability of the proposed strategy, we use 1 or 2 P-FOAMs (from ±0.8 to ±2.2 at the step of 0.2) to conduct the high-dimensional data transmission, which enables a total of $C_{8}^{1}+C_{8}^{2}=$ 36 levels information (log_2_36 ≈ 5.17 bits) including 10 Arabic numerals (0 to 9) and 26 English letters (A to Z) (Table S1 in Note S6). For example, the letter “N” contains 2 P-FOAMs of ±1.2 and ±1.8. Hence, the cross-sectional maps of transmitting letters “NJNU” are simulated and presented in Fig. 3A. More importantly, despite experiencing severe distortions by turbulence, the characteristics of phase dislocations corresponding to pressure valleys are still preserved in Fig. 3B. Meanwhile, the experimental maps of pressure and phase in the transverse plane in Fig. 3C agree well with the simulations in Fig. 3B in terms of shape and size. Therefore, the simulated cross-sectional maps under turbulences can be applied to approaching the nonideal realistic transmission condition. Notably, the proposed strategy exempts from the heavy burden of the high-precision scanning measurement in 2 dimensions and the experimental training set to a great extent.

On the basis of the phase-dislocation-mediated recognition of P-FOAMs using the CNN, the transmitting letters “NJNU” are classified using the 16-point single-ring sampling, and the corresponding probability distribution as the function of the class is plotted in Fig. 3D. After 50 iterations, the classification accuracy reaches 97.55%, and the loss decreases quickly to zero. The probability of recognizing letters “N” and “J” being misread as “H” and “F” is nearly 15%, which may be resulted from the large TC gap between the P-FOAMs of ±1.2/±1.8 and ±1.0/±2.0. It means that the circular sparse sampling with *r* = 17 mm cannot adequately acquire the featured characteristics of phase dislocations for FAVs with different P-FOAMs. Moreover, the stability of data decoding can be evaluated quantitatively by the standard deviation (SD) of $\sigma =\sqrt{\sum {\left(P_{m}^{\prime }-P_{m}\right)}^{2}/K}$ [31], where *K* is the number of information level and *P_m_* and $P_{m}^{\prime }$ denote the experimental and numerical probabilities, respectively. The SDs of transmitting and decoding letters “NJNU” are about 1.34%, 2.23%, 4.27%, and 0.17%, respectively. Two methods, i.e., the 32-point single-ring sampling at *r* = 17 mm and the 32-point dual-ring sampling at *r* = 13.5 and 20.5 mm (corresponding to vortex radii for *l* = ±1 and ±2), are proposed to achieve a more stable decoding. As can be seen from Fig. 3E and F, the classification accuracies achieved by such 2 sampling methods reach 99.15% and 99.88% after 50 iterations with the training time cost of about 11 min. The slightly reduced SD of the 32-point single-ring sampling indicates that, once the sampling point meets the resolution requirement of the phase-dislocation-mediated theory in Fig. 2C, the stability and security of FAV communications cannot be greatly improved by only increasing the sampling point. The SD of recognizing letters “NJNU” using the 32-point dual-ring sampling can be quickly reduced to zero. It is also reasonable to draw that the application of the dual-ring sparse sampling can fully extract and identify the azimuth of phase dislocation, so as to enhance the recognition accuracy of FOAMs in the multiplexed high-dimensional communication. Then, nonideal transmission conditions with gradient turbulences, angular deflections, translations, and radial rotations are simulated to evaluate the performance of the FOAM communication strategy. The corresponding cross-sectional distributions and decoding accuracies in Note S7 indicate that our strategy has a good anti-interference capability when the nonideal transmission factors are not so serious.

**Fig. 4.**
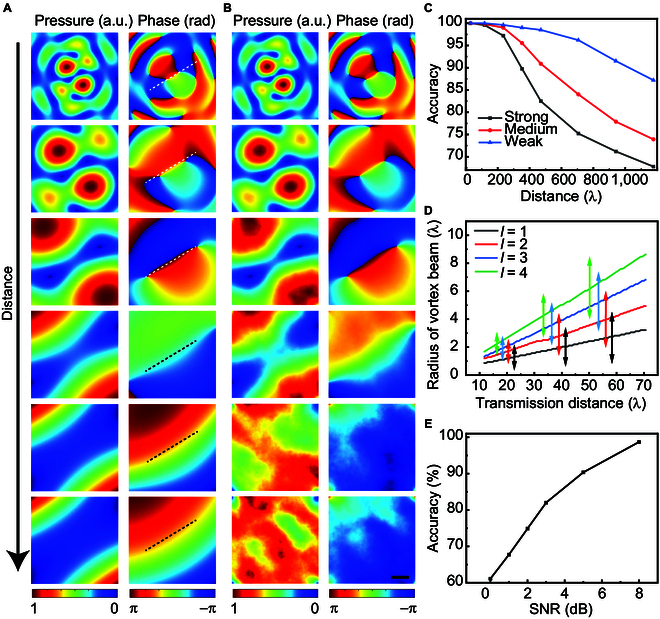
Effect of transmission distance, turbulence, and noise on FOAM decoding. (A) Cross-sectional maps of pressure and phase at different transmission distances of 29.41λ, 58.82λ, 117.64λ, 235.29λ, 588.24λ, and 1,176.47λ in the same field of view for *l* = ±1.4. (B) Corresponding cross-sectional maps of pressure and phase in the nonideal transmission condition with a weak turbulence of $C_{n}^{2}=10^{-14}$. As indicated by the dashed lines, the azimuthal angle of phase dislocation is independent of the transmission distance. (C) Distance dependence of the decoding accuracy with various turbulences (strong $C_{n}^{2}=10^{-12}$, medium $C_{n}^{2}=10^{-13}$, and weak $C_{n}^{2}=10^{-14}$) for the 32-point dual-ring sampling. (D) Distance dependences of the optimized sampling radius for coupled FAVs with P-FOAMs of *l* = ±1 to ±4. (E) Decoding accuracy distribution with respect to the SNR of Gaussian noise. Scale bar, 2 cm.

In addition, we also study the effect of transmission distance on FOAM decoding with the turbulence changing from weak to strong, maintaining the sampling radii of *r* = 13.5 and 20.5 mm. From the cross-sectional pressure and phase maps at various transmission distances as shown in Fig. 4A, it can be seen that although the size of the coupled FAV expands as the increased in the distance, the azimuth of phase dislocation remains unchanged. The comparison between the P-FOAM and single-FOAM fields in Note S8 demonstrates that the P-FOAM has a stable phase dislocation with the azimuth being independent of the transmission distance, while single FOAM does not have. Moreover, phase dislocations are preserved even when the sampling radius is smaller than the vortex radius, which provide striking features for stable decoding. Under nonideal conditions, the acoustic pressure attenuates accordingly as the propagation distance extends, and the acoustic distribution will be influenced by a stronger turbulence, resulting in a gradually increased distortion with a poorer signal-to-noise ratio (SNR) as shown in Fig. 4B. Subsequently, the decoding accuracy decreases as the increase in transmission distance as shown in Fig. 4C. Taking the decoding accuracy of 95% as the criterion, the transmission distances can reach about 270λ, 365λ, and 765λ for the strong, medium, and weak turbulence, respectively. Hence, the decoding accuracy can be optimized by selecting an appropriate sampling radius for AVs of different orders at various transmission distances according to the relationships in Fig. 4D. Moreover, the divergence [32] can still be further alleviated through the trade-off between the channel capacity and the transmission distance. Furthermore, the noise level effect on the accuracy evaluation is performed and shown in Fig. 4E, and it shows that the decoding accuracy of 95% can still be achieved under the SNR of 6 dB, demonstrating the high robustness of the proposed technology.

### Ultrahigh dimension of FOAM decoding

**Fig. 5.**
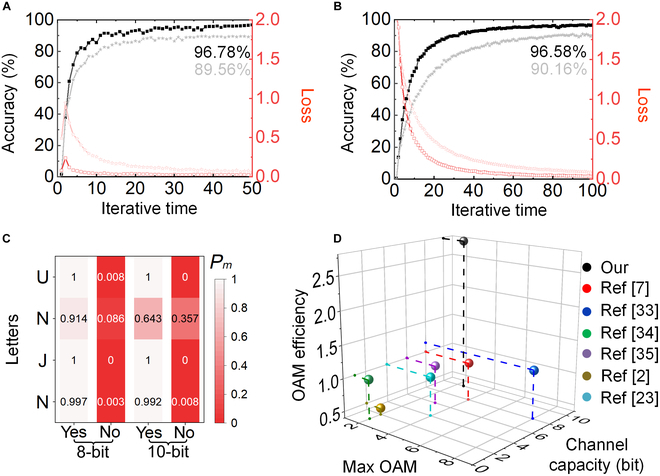
Recognition accuracy (black solid squares) and loss (red hollow squares) for training. The results are achieved for the (A) 8-bit and (B) 10-bit P-FOAM-multiplexed communications based on the 32-point dual-ring sampling, and the curves for the conventional FOAM-multiplexed communication are provided for reference in gray solid and red hollow stars. (C) Experimental recognition probabilities of the 8-bit and 10-bit channels for transmitting letters “NJNU”, where “yes” mean that the decoding is true, and vice versa. (D) Comparison between our work and other milestone works in respect of the max OAM, the channel capacity (bit), and the OAM efficiency.

Since there is no TC limit of FOAM multiplexing in mathematics, P-FOAMs hold a great promise of infinitely expanding the dimension of data transmission in theory and hence achieve an ultrahigh channel capacity of information transmission. To further boost the channel capacity and demonstrate the transmission reliability, the 8-bit (*l* = ±0.8 to ±2.2 at the step of 0.2) and 10-bit (*l* = ±0.6 to ±2.4 at the step of 0.2) P-FOAMs are used to record the total information levels of $\sum_{n=1}^{8}C_{8}^{n}=255$ and $\sum_{n=1}^{10}C_{10}^{n}=1023$, respectively. Then, the recognition accuracy and loss for training based on the 32-point dual-ring sampling for the 8-bit and 10-bit data transmissions using the P-FOAM-multiplexed FAVs are illustrated in Fig. 5A and B, respectively. The accuracies of 96.78% and 96.58% for the P-FOAM-multiplexed FAVs are much higher than those (89.56% and 90.16%) of the conventional FOAM-multiplexed ones. The loss of the 8-bit case reduces to zero after 10 iterations, which is about 5 times faster than that of the conventional one using the FOAM. The results demonstrate the significance of the phase-dislocation-mediated method for the FAV communication. The excellent anti-interference performance of the 8-bit and 10-bit channels is also proved by the final accuracy of more than 96.5% after 50 and 100 iterations. By applying the CNN to the experimental data of transmitting letters “NJNU” acquired by the dual-ring sampling (Table S2 and Note S6), the decoded results are clearly displayed in Fig. 5C. With the exception of the relatively lower probability (0.64) induced by the increased classifications and experimental errors, all probabilities of decoding the other letters exceed 0.90 and the second letter “N” can be recognized successfully. Therefore, we can induce that a high-accuracy and high-dimensional information transmission can be realized in a small OAM scope. In addition, as shown in Fig. 5D and Note S9, the 10-bit channel within a small OAM scope of ±0.6 to ±2.4 leads to a greatly improved OAM efficiency (defined as channel capacity/OAM scope) of about 2.78, compared with those of the other milestone works [33–35]. Hence, benefiting from the extra advantages of the easy data coding/decoding in high-dimensional AV communications, our work is experimentally applicable using a simplified transceiver system with less divergent effects of traditional AV beams.

### Ultrahigh-resolution of FOAM recognition

**Fig. 6.**
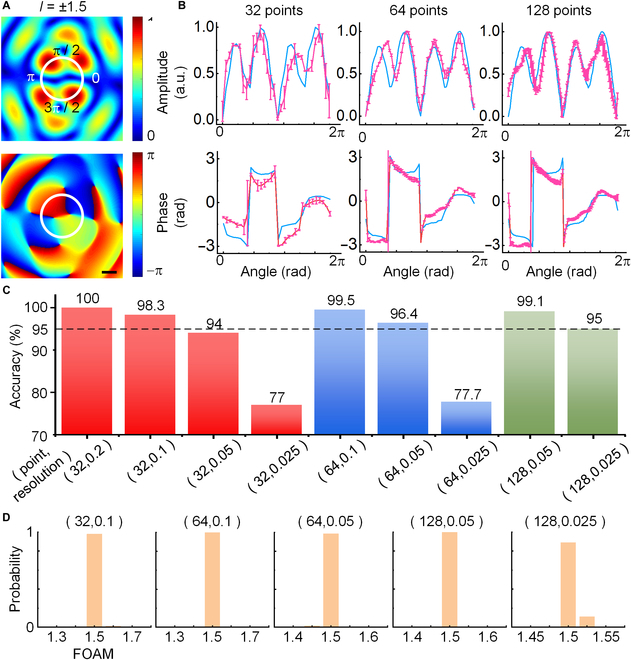
Recognition potential of FOAMs for the phase-dislocation-mediated CNN based on the single-ring sampling. (A) Cross-sectional distributions of pressure and phase for the P-FOAM of *l* = ±1.5. (B) Numerical and experimental circular pressure profiles acquired by the sing-ring sampling of 32, 64, and 128 points with *r* = 17 mm, with the error bars obtained by more than 2 groups of experiments. (C) Training accuracy with various sampling points and FOAM resolutions. The successful decoding of the P-FOAM for the training accuracy higher than 95% with the corresponding probabilities are shown in (D). Scale bar, 1 cm.

To discuss the feasibility of the resolution limitation of FOAMs, we conduct the study based on the single-ring sparse sampling with the FOAM scope from ±1.0 to ±2.0. Taking the P-FOAM of *l* = ±1.5 as an example, the cross-sectional maps of pressure and phase are simulated in Fig. 6A. The numerical and experimental profiles of pressure and phase in Fig. 6B acquired by the single-ring sampling of 32, 64, and 128 points with *r* = 17 mm show a better consistency for more sampling points. Furthermore, the holographic images for various sampling points are considered as the input of CNN for training to classify P-FOAMs with the resolutions of 0.2, 0.1, 0.05, and 0.025. The accuracy and loss for training as functions of the sampling number and the P-FOAM resolution are shown in Fig. 6C, with the decoding probability distribution shown in Fig. 6D (the training process is shown in Note S10). Taking 95% as the criteria, the FOAM resolutions of 0.1, 0.05, and 0.025 for the respective sampling points of 32, 64, and 128 consist well with the predictions in Fig. 2C. It indicates that the recognition accuracy increases with the increase in the sampling number, while decreases with the increase in the FOAM resolution.

For the relevant frontier in optics, the high-precision recognition of OAMs was realized by the deep learning on the basis of thousands of camera recorded intensity pictures with the down sampled pixels [20] from 1,920 × 1,080 to 224 × 224. In this study, we demonstrate the superiority of the infinite channel capacity expansion for the phase-dislocation-mediated FAV communication and realize the ultrahigh resolution (0.025) of P-FOAMs with the high accuracy of 95% based on the 128-point single-ring sampling. Although the P-FOAM resolution is affected by the sampling resolution, the nonideal condition and the CNN, we can further improve the accuracy by introducing the experimental datasets, the dual-ring sampling and the superior CNN architecture, exhibiting crucial potentials for the optimized performance of FAV communications.

## Discussion

This article mainly focuses on the principle of information transmission and recognition for the multiplexed P-FOAMs and pays relatively little attention to the communication performance because the communication speed and bit error rate belong to the technical category. Although the transmission rate is still limited by the circular sparse sampling, we can achieve the real-time decoding using a simplified ring array of receivers in the future, which was proposed by Shi et al. [23]. As a counterpart, we have achieved 4-fold information level with fewer receivers and less divergence. The maintained phase dislocation with P-FOAMs (independent of the transmission distance and the sampling radius) in a constrained TC scope can greatly prevent the divergent effect of high-order AVs, and the restricted measurement radius is also beneficial to the system design even in the long-distance transmission. In addition, the accuracy and resolution of FOAM recognition can also be further improved by introducing the experimental data into a superior neural network as the training set.

We have rigorously demonstrated the method’s potential to enable an infinite expansion of channel capacity in AV communication. This technique can seamlessly integrate with both the frequency-division and time-division multiplexing strategies, enhancing its versatility and applicability. Importantly, the method’s scope transcends the boundaries of the acoustic domain, and its adaptability can extend to the electromagnetic domain through opposite TCs and machine learning. Our method also presents a range of other applications except for the FOAM communication. Primarily, using FAVs with opposite TCs holds significant promise for achieving enhanced azimuthal resolution in imaging with the natural characteristics to be composed into a series of OAM modes [36]. In addition, FAVs offer inherent multi-OAM capabilities and provide a unique avenue to disentangle the longitudinal and angular accelerations of objects [37], hence showcasing the method’s practical relevance and transformative potential.

In conclusion, we propose and realize a phase-dislocation-mediated high-dimensional FAV communication strategy for the first time in acoustics. The property of the stable phase dislocation can be fully extracted by the holographic signals of circular sparse sampling, promoting the breakthrough from theory to practice. The high-dimensional FAV communication with P-FOAMs in a constrained TC scope not only drastically improve the OAM efficiency but also greatly prevent the divergent effect of high-order AV beams. Advantages of “physics” and “intelligence” are demonstrated by the high accuracy, high robustness, and high speed for the high-capacity channels. The infinitely expanded channel capacity is further verified by the enhanced FOAM resolution of 0.025. Our technology reaches 3-fold OAM efficiency, 4-fold information level, and 5-fold OAM resolution, which may fill the gaps in FAV communications.

## Materials and Methods

### Machine learning

To improve the real-time and automation performance of the high-dimensional data communication, holographic images of pressure and phase of the P-FOAM-multiplexed FAVs are achieved by the circular sparse sampling and then used as the input of CNN for training. Our phase-dislocation-mediated high-dimensional FAV communication strategy based on CNN was constructed using PyCharm and trained in a desktop with an I9-10900K CPU and an RTX3080 GPU (Intel I9-10900K CPU @ 3.07 GHz, GPU: NVIDIA GeForce RTX3080 GPU, RAM: 32 GB and ROM: 2.5 TB), whose architecture is shown in Note S11. The learning rate is 0.0001 (8-bit and 10-bit channels) or 0.001 (other). The cross-entropy is used to calculate the loss function, and the optimizer is Adam. The network mainly includes the CNN layers of convolution, lower sampling, full connection, and output. To analyze the performance of anti-interference and the stability of FOAM recognition, the influence of the realistic transmission channels, i.e., angular deflection (−1.5° to 1.5°), translation (−1.5 to 1.5 mm), radial rotation (−1.5° to 1.5°) of the transmitter array, atmosphere turbulence ($C_{n}^{2}=10^{-12}$), were considered as shown in Note S7. Then, 400 and 100 images of training and testing of the coupled FAVs with opposite TCs were simulated with MATLAB programming, which are used as the input of CNN for training.

### Experiment system setup

To verify the proposed mechanism, we built an experimental system of the phase-dislocation-mediated FAV communication with circular sparse sampling as shown in Note S2. Sixteen ultrasonic transducers (NU40C10T-2, Porcelain Technology, China) with a radius of 2 mm and a center frequency of 40 kHz were uniformly fixed on an acrylic disk (radius *a* = 50 mm) to form the single-ring array of transmitters. A field programmable gate array (Altera Cyclone IV, Altera Corporation, USA) was utilized to generate 16 square waves at the frequency of 40 kHz with adjustable phases. After filtered by low-pass filters and amplified by power amplifiers (OPA552, Texas Instruments, USA), 16 phase-controllable cosine signals were sent out to excite the source array to construct P-FOAM-multiplexed FAVs in free space. An ultrasonic transducer (NU40C10T-2, Porcelain Technology, China) was used as the receiver to detect the acoustic pressure around the sampling circle. Moved by the 2-dimensional motion stage and collected by the digital oscilloscope (Agilent DSO9064A, Agilent Corporation, USA), the single/dual-ring sampling of *N* points in the transverse plane at *z*_0_ = 29.41λ was accomplished. In experiments, the surface vibration velocity of the transducer was calibrated to *A* = 60 mm/s, and the sound velocity and density in the air were set to *c*_0_ = 340 m/s and *ρ*_0_ = 1.225 kg/m^3^, respectively.

## Acknowledgments

**Funding:** This work was supported by the National Natural Science Foundation of China (grant nos. 11934009, 11974187, 12174198, 12227808, 62025501, 31971376, and 92150301), National Key R&D Program of China (2022YFC3401100), the Natural Science Foundation of Jiangsu Province (no. BE2022814), and the Qing Lan Project of Jiangsu Province, China.

**Author contributions:** R.C., G.G., Y.H., D.Z., P.X., and Q.M. conceptualized the ideas. R.C., G.G., Y.H., J.T., and Q.M. studied the methods. R.C., G.G., P.X., and Q.M. did the investigation. R.C. and Y.H. wrote the software. R.C., W.Y., Y.H., X.L., C.K., Y.L., and G.G. did the experiment. R.C., G.G., J.T., P.X., and Q.M. wrote the article. R.C. and G.G. did the visualization. G.G., J.T., D.Z., P.X., and Q.M. acquired the funding.

**Competing interests:** The authors declare that they have no competing interests.

## Data Availability

Data and code can be obtained from authors through reasonable requirements.

## Supplementary Materials

Supplementary 1 Notes S1 to S11
Figs. S1 to S8
Tables S1 to S3

Supplementary 2 Movie S1
