# Supplementary material for: Phase-Dislocation-Mediated High-Dimensional Fractional Acoustic-Vortex Communication
Source: Research (Wash D C). 2023 Dec 1;6:0280. doi: 10.34133/research.0280 (PMC10907020; doi:10.34133/research.0280)
Supplement: Supplementary 1 — Notes S1 to S11 Figs. S1 to S8 Tables S1 to S3 [file research.0280.f1.docx]

Supplementary Materials

**Phase-dislocation Mediated High-dimensional Fractional Acoustic-vortex Communication**

Ruijie Cao^1,2,4^,**^†^** , Gepu Guo^1,*^,**^†^**, Wei Yue^1^, Yang Huang^1,3^, Xinpeng Li^1^, Chengzhi Kai^1^, Yuzhi Li^1^, Juan Tu^3^, Dong Zhang^3*^, Peng Xi^2,4*^ and Qingyu Ma^1*^

^1^ School of Computer and Electronic Information, Nanjing Normal University, Nanjing 210023, China

^2^ Department of Biomedical Engineering, College of Future Technology, Peking University, Beijing 100871, China

3 Institute of Acoustics, Nanjing University, Nanjing 210093, China

4 National Biomedical Imaging Center, Peking University, Beijing 100871, China

**^†^** These authors contributed equally to this work

*Correspondence: G.G. ([guogepu@njnu.edu.cn](mailto:guogepu@njnu.edu.cn)), D. Z. ([dzhang@nju.edu.cn](mailto:dzhang@nju.edu.cn)), P.X. ([xipeng@pku.edu.cn](mailto:xipeng@pku.edu.cn)), Q.M. ([maqingyu@njnu.edu.cn](mailto:maqingyu@njnu.edu.cn))

**Content**

[Supplementary Note 1: Principle of P-FOAM multiplexing in realistic circumstances 1](#_Toc146986860)

[Supplementary Note 2: Experimental system of FAV communication 4](#_Toc146986861)

[Supplementary Note 3: Construction of coupled FAVs with opposite TCs 5](#_Toc146986862)

[Supplementary Note 4: Coupled FAVs with the FOAM resolution of 0.2 7](#_Toc146986863)

[Supplementary Note 5: Training and decoding of non-multiplexed P-FOAM 8](#_Toc146986864)

[Supplementary Note 6: Data encoding and look-up table 9](#_Toc146986865)

[Supplementary Note 7: Influence of non-ideal communication channels 10](#_Toc146986866)

[Supplementary Note 8: Comparison between the P-FOAM and single-FOAM beams 12](#_Toc146986867)

[Supplementary Note 9: Comparison of different AV communication techniques in respect of the capacity and OAM range 13](#_Toc146986868)

[Supplementary Note 10: Curves of accuracy and loss with various sampling points and FOAM resolutions 14](#_Toc146986869)

[Supplementary Note 11: Architecture of CNN 15](#_Toc146986870)

# Supplementary Note 1: Principle of P-FOAM multiplexing in realistic circumstances

In order to construct the coupled FAV, a single-ring array of *N* transducers uniformly distributed on a circumference (radius *a*) with the spatial angle difference of is established in **Fig. 1**. The initial phases of $\pm\text{ }2\pi l(n-1)/N$ are used to drive the *n*-th source (azimuthal angle ) to construct two coxial FAV beams with opposite fractional TCs ( ± *l* ) in free space. According to the theory of point source radiation, the coupled FAV beam generated by the interaction of two AV beams of ±*l* orders can be calculated by [7]

$p(r,\phi,z\text{，}\pm l)=A\frac{jk\rho_{0}c_{0}}{2\pi}\sum_{n\text{=1}}^{N} \left[\frac{\exp[j\left(-kR_{n}+l\phi_{n}\right)]}{R_{n}}+\frac{\exp[j\left(-kR_{n}-l\phi_{n}\right)]}{R_{n}}\right]$. (1)

where is the distance between the observation point and the *n*-th source *M_n_*, *A* is the source particle velocity, $k=\omega/c_{0}$is the wave number with $c_{0}$ and $\rho_{0}$ being the acoustic speed and the density of the media, $\omega$ is the angular frequency of the system.

As is reported, the FOAM expansion can be described by the summation of infinite integer-order OAMs as [15, 28], where *m* represents the OAM of integer-order. By substituting the formula into Eq. (1), the acoustic pressure of the coupled FAV beam with the opposite TCs of ±*l* can be revised to

. (2)

Where denotes the acoustic pressure of the traditional AV of *m*-th integer-order, which has the annular pressure distribution with a phase spiral around the center. The cross-sectional pressure map of the the coupled FAV at $z=z_{0}$ can be described by $p(r,\phi,z_{0},m)=A\left(r,m\right)\exp\left[j\gamma\left(r,m\right)\right]\exp(jm\phi)$[26], where $A\left(r,m\right)$ and $\gamma\left(r,m\right)$ represent the TC dependent distributions of pressure and phase along the radial direction, and satisfy $A\left(r,m\right)=A\left(r,-m\right)$ and $\gamma\left(r,m\right)=\gamma\left(r,-m\right)$. So the coupled FAV can be further simplified to

$p\left(r,\phi,z_{0},\pm l\right)=\frac{\sin\left(\pi l\right)}{\pi}\sum_{m=-\infty}^{\infty} \frac{2\exp\left[j\gamma\left(r,m\right)\right]A\left(r,m\right)}{l-m}\cos\left(m\phi+\pi l\right)$. (3)

It shows that, the coupled FAV can be analogously described as the superposition of the coupled AVs of integer-orders with the weight of $\sin\left(\pi l\right)\text{/}\pi\left(l-m\right)$. Meanwhile, the azimuthal angle of the phase-dislocation rotates by $l\pi/m$ relative to that of the coupled AV of the *m*-th integer-order, which provides a stable distinctive feature for the recognition of FOAMs in principle.

In order to realize the synchronous communication of high-dimensional data, we build a P-FOAM-multiplexed FAV with *S* pairs of FOAMs. However, since the distributions of pressure and phase may be distorted as propagating in the realistic channel, a random phase screen based on the power spectrum with a negligible thickness is introduced to simulate the influence of actual environments, which can be simplified by the random complex matrix *C* with the real and imaginary variances of 1 and the mean value of 0. Thus, the acoustic pressure of the P-FOAM-multiplexed FAV is achieved as [30]

$p_{S}(r,\phi,z_{0})=\exp[j\psi(x,y)]\sum_{s=1}^{S} \left[p(r,\phi,z_{0},\pm l_{S})\right]$, (4)

where *s* is the number of the P-FOAM, $\psi\left(x,y\right)=F^{-1}\left\{C\times\sigma\left(k_{x},k_{y}\right)\right\}$represents the random phase with the inverse Fourier transform $F^{-1}\left\{\cdot\right\}$, $\sigma(k_{x},k_{y})=\frac{2\pi}{N\Delta x}\sqrt{\Phi(k_{x},k_{y})}$ denotes the variance of the spectral power, and $\Delta x$ is the sampling interval of the phase screen. $\Phi(k)\text{=}2\pi\left(k_{x}^{2}+k_{y}^{2}\right)\Delta z\Phi_{n}\left(k_{x},k_{y}\right)$ decribes the phase screen with $k_{x}$ and $k_{y}$ being the coordinates in the frequency domain, $\Phi_{n}(k)=0.033C_{n}^{2}k^{-11/3}$with the beam number *k* and the refractive index $C_{n}^{2}$ of the atmospheric structure. Although the pressure distribution of the P-FOAM-multiplexed FAV is complex, the rotation characteristics of the phase-dislocation determined by the P-FOAM still retains.

# Supplementary Note 2: Experimental system of FAV communication


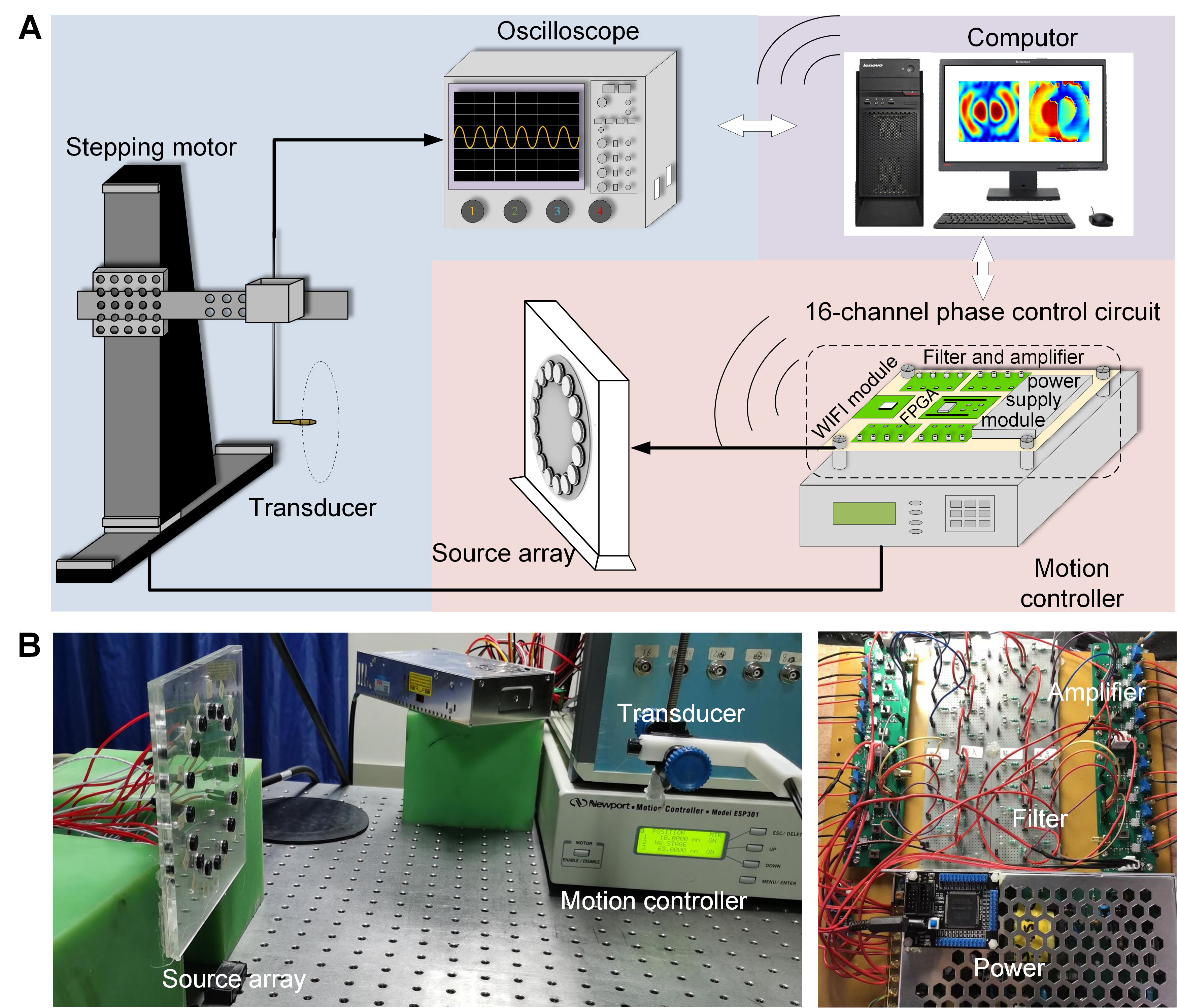


**Fig. S1.** **Experimental system. (A)** Block diagram of the experimental system for the phase-dislocation mediated FAV communication based on circular sparse sampling, including the P-FOAM-multiplexed FAV construction (pink background), the single-ring sparse sampling (blue background), and the recognition and decoding of FOAMs (purple background). **(B)** Photographs of the single-ring transceiver arrays, the home-made 16-channel phased driving circuit, and the high-precision 2D scanning system.

# Supplementary Note 3: Construction of coupled FAVs with opposite TCs

The fractional-order TC can be described by $\text{l}=\text{L+}\mu$, where $\text{L}=\left\lfloor\text{l}\right\rfloor$ and $\mu=\text{l}-\text{L}$ represent OAMs of the integer and fractional orders. As can be seen from Fig. S2, for $\mu=0$ and *L* = 1, AVs with the positive and negative TCs show clear annular pressure distributions and opposite phase spirals around the vortex center (pressure null). After the interaction of the AVs with opposite TCs, a stable pressure distribution with 2*L* petals and 2*L* phase-dislocations can be observed. As $\mu$ is increased to 0.2, the phase singularities at the center of FAVs with the TCs of ± *l* moves to the first and third quadrants, and hence break the annular profiles for AVs of integer orders to form a uniform distribution. However, the coupled AV is still divided into two symmetrical parts by a low pressure stripe with a clockwise rotation of about 20° ($\pi/9$). When $\mu$ is increased to 0.5, the offset of the initial phase singularity reaches its maximum and produces an obvious pressure discontinuity. In addition, due to the phase singularity splitting, a new unit AV emerges in the opposite offset direction of the phase singularity. Then, a crescent-shaped low-pressure region is formed by the continuous clockwise rotation of the phase-dislocation in Fig. S2. For a further increased $\mu$, the new phase singularity converges gradually toward the origin, and the crescent-shaped low-pressure region shrinks to a low-pressure point. For the interaction of the FAVs with opposite TCs, the cross-sectional pressure map of the coupled FAV is divided into 2 (*L*+1) petals with a further clockwise rotation of the phase-dislocation. Finally, as $\mu$ increases to 2, the symmetrically distributed uniform petals with 2(*L*+1) phase-dislocations are created by the coincident phase singularities.

Compared with the FAVs with a single TC, the phase-dislocation of the coupled FAV is more obvious. Taking the radial pressure distributions of FAVs with *l* = 1.0 and 1.2 as examples, the subtle non-uniform annular distribution and phase singularity migration are not obvious enough to recognize the inlcuded FOAMs precisely. However, due to the interaction of two FAVs with opposite TCs, the almost unchanged radial distributions of pressure and phase-dislocation at different propagation distances embody the excellent performance of anti-interference. Moreover, for the FOAM increase of 0.2, the azimuthal rotation of the phase-dislocation reaches 30° in the clockwise direction, which provides a enough angle difference for the recognition of FOAMs.


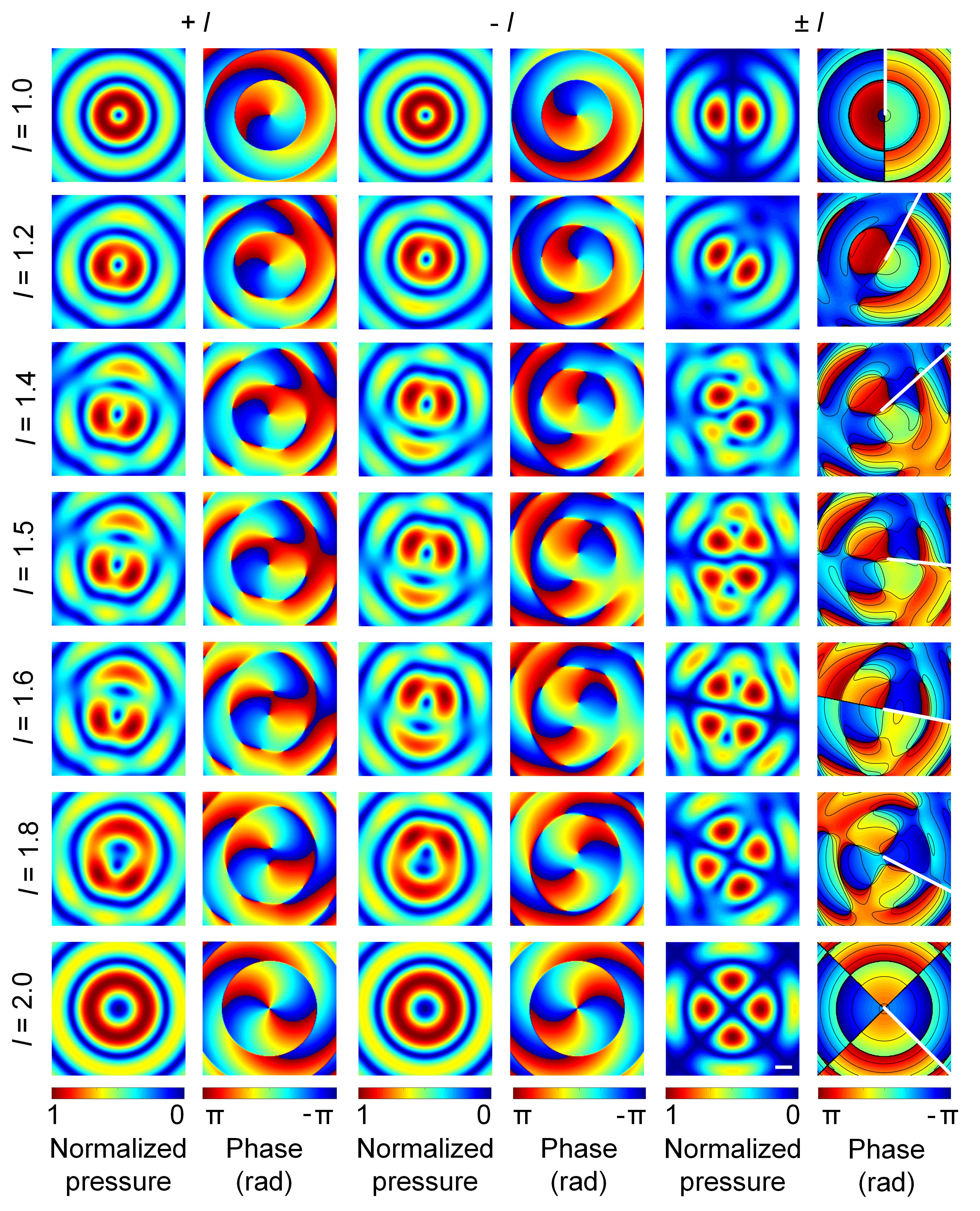


**Fig. S2**. **Construction of coupled FAVs of different orders**. Cross-sectional distributions of pressure and phase at *z*_0_ = 29.41λ with the TCs of *l*, - *l*, and ± *l*. The white line represents one of the phase-dislocation. Scale bar: 1 cm.

# Supplementary Note 4: Coupled FAVs with the FOAM resolution of 0.2


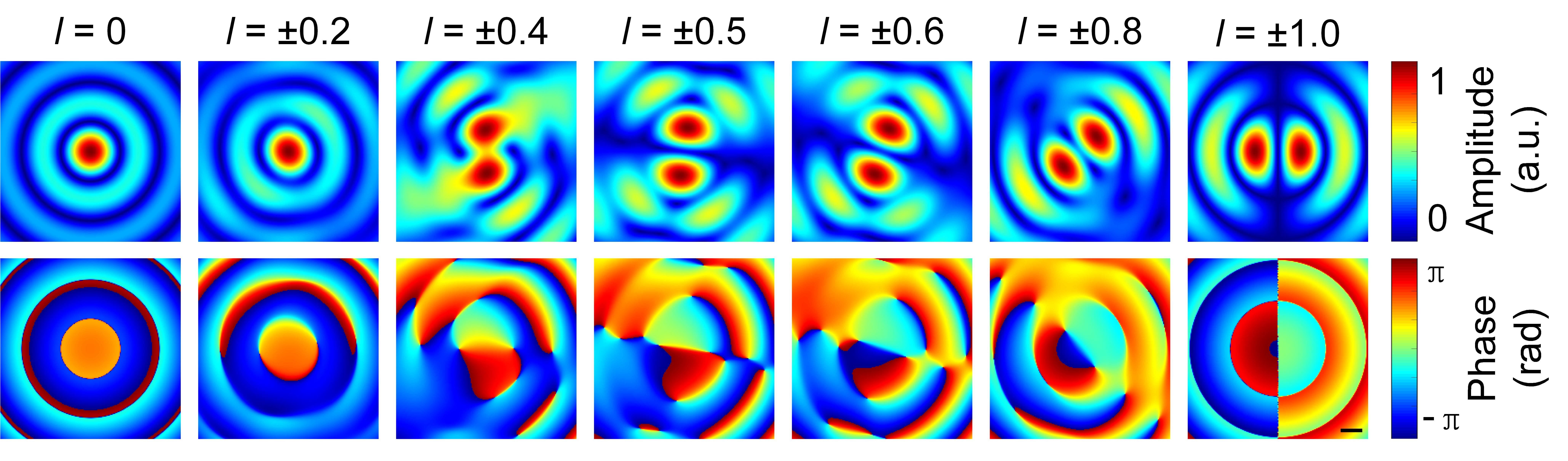


**Fig. S3**. **Cross-sectional distributions of coupled FAVs with the FOAM resolution of 0.2.** The coupled FAVs are constructed with the opposite FOAMs of 0 ~ ±1.0**.** Scale bar: 1 cm.

# Supplementary Note 5: Training and decoding of non-multiplexed P-FOAM


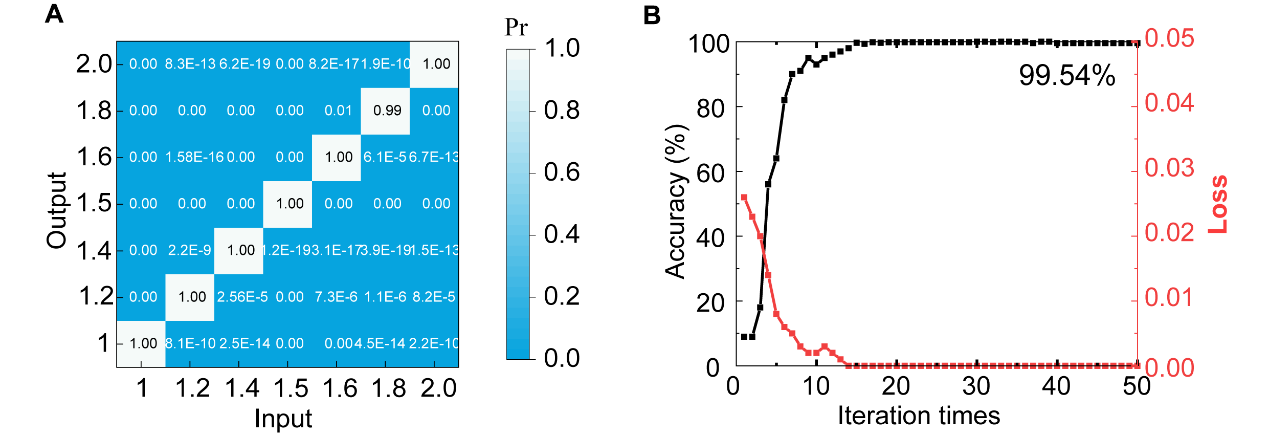

**Fig. S4.** Training and decoding results of single P-FOAM. (**A**) Decoding results of probability for *l* = ±1.0 ~ ±2.0, and (**B**) training curves of accuracy and loss for FOAM decoding using the 16-point single-ring sampling.

# Supplementary Note 6: Data encoding and look-up table

**Table S1. Data encoding and look-up table for the 5.17-bit channel**

| ***l*** | **±0.8** | **±1** | **±1.2** | **±1.4** | **±1.6** | **±1.8** | **±2.0** | **±2.2** | **Class** |
| --- | --- | --- | --- | --- | --- | --- | --- | --- | --- |
| **j** | 0 | 1 | 0 | 0 | 0 | 0 | 1 | 0 | 20 |
| **n** | 0 | 0 | 1 | 0 | 0 | 1 | 0 | 0 | 24 |
| **u** | 0 | 0 | 1 | 1 | 0 | 0 | 0 | 0 | 31 |

**Table S2. Data encoding and look-up table for the 10-bit channel**

| ***l*** | **±0.6** | **±0.8** | **±1** | **±1.2** | **±1.4** | **±1.6** | **±1.8** | **±2.0** | **±2.2** | **±2.4** | **Class** |
| --- | --- | --- | --- | --- | --- | --- | --- | --- | --- | --- | --- |
| **j** | 0 | 0 | 1 | 0 | 0 | 0 | 0 | 1 | 0 | 0 | 32 |
| **n** | 0 | 0 | 0 | 1 | 0 | 0 | 1 | 0 | 0 | 0 | 37 |
| **u** | 0 | 0 | 0 | 1 | 1 | 0 | 0 | 0 | 0 | 0 | 46 |

# Supplementary Note 7: Influence of non-ideal communication channels


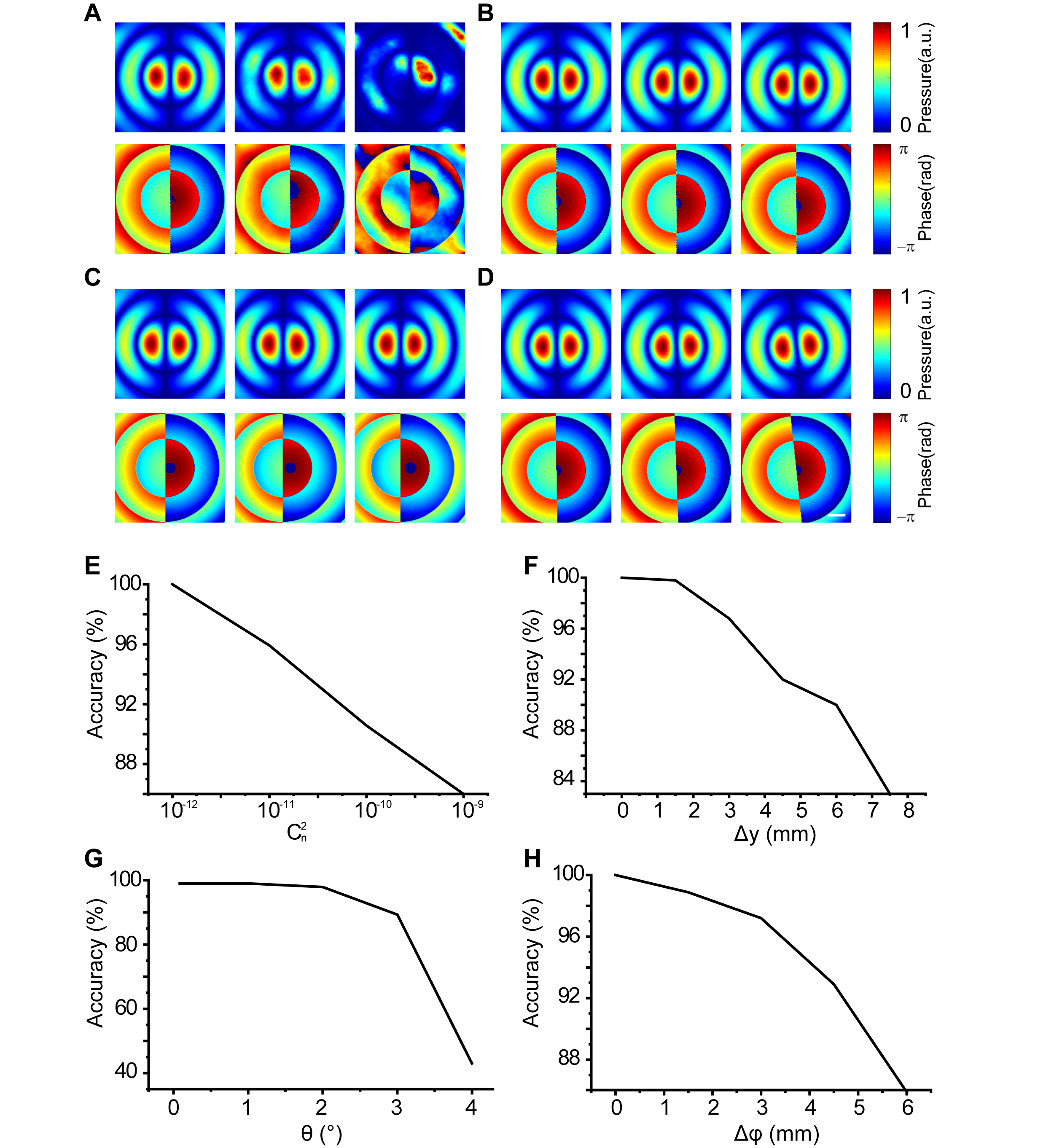


**Fig. S5.** **Influence of non-ideal realistic communication channels.** Cross-sectional maps of pressure and phase with the **(A)** turbulence of *C_n_*^2^=10^-13^~10^-11^**, (B)** translation of ∆*y* = 2 ~ 6 mm, **(C)** angular deflection of θ = 2° ~ 6°, and **(D)** radial rotation of ∆ φ = 2° ~ 6°. Corresponding decoding accuracies as functions of the **(E)** gradient turbulence, (**F**) translation, (**G**) angular deflection, and (**F**) radial rotation. Scale bar: 1cm.

The cross-sectional distributions of pressure and phase with different gradient turbulences, angular deflections, translations, and radial rotations are simulated as shown in **Fig. S5 (A, B, C, D)**. It is clear that the field distortion becomes prominent with the increase of turbulence. The misalignments of the angular deflection, translation, and radial rotation also produce the field translation and rotation. Those non-ideal realistic factors cause the decrease in decoding accuracy in **Fig. S5 (E, F, G, H)**. Simulation results prove that this method has a good anti-interference capability overall, while the decoding accuracy is lower than desired for severe non-ideal transmission conditions.

# Supplementary Note 8: Comparison between the P-FOAM and single-FOAM beams


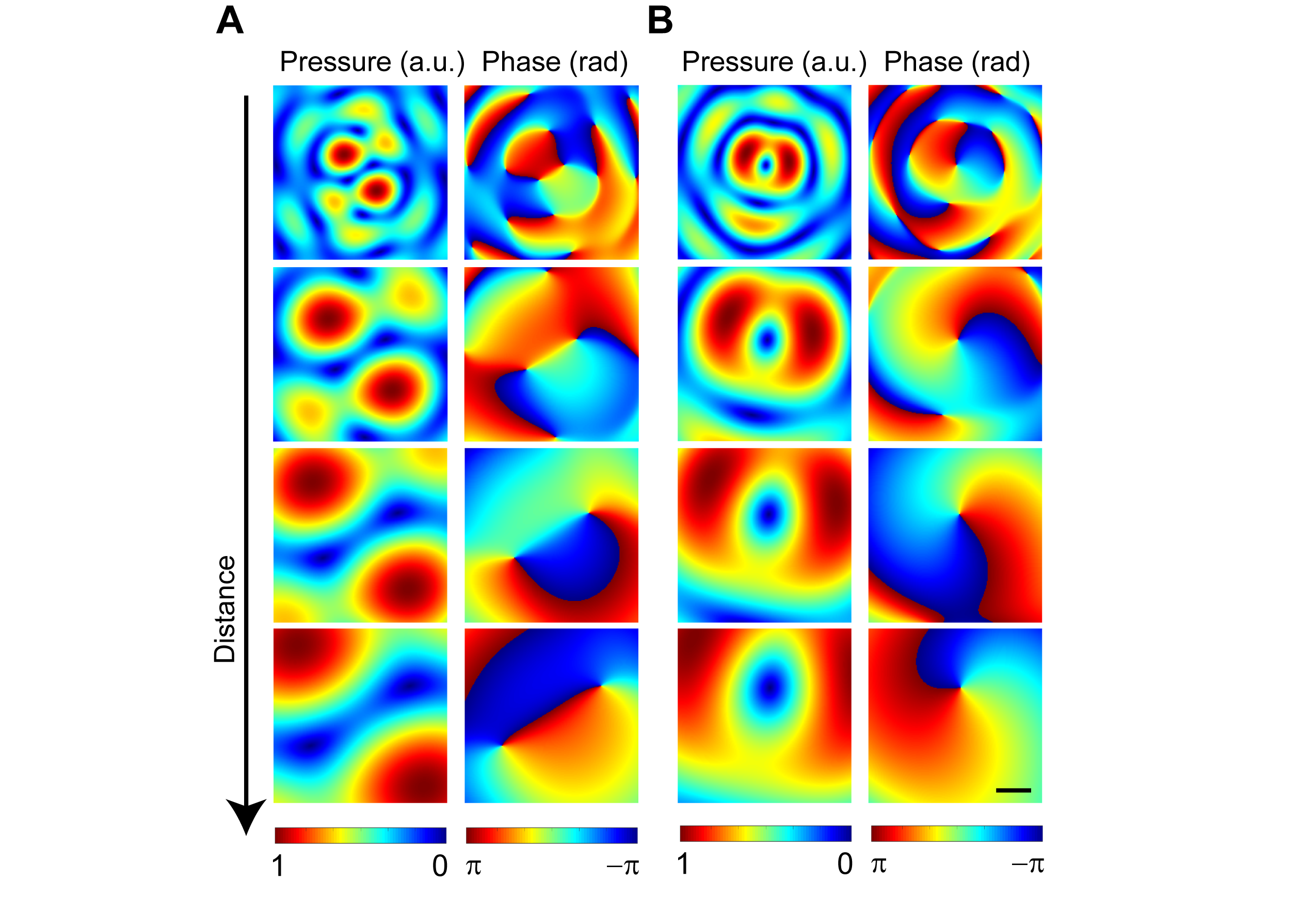


**Fig. S6.** **Cross-sectional maps of FAV beams at different transmission distances.** (**A**) P-FOAM of *l* = ±1.4 and (**B**) single-FOAM of *l* = 1.4. Scale bar: 2 cm.

The FAV beam with the single-FOAM of *l* = 1.4 has an obviously rotated phase-dislocation as the increase of the transmission distance, indicating that the decoding depends on the transmission distance. While, the FAV beam with the P-FOAM of *l* = ±1.4 has a stable phase-dislocation with an almost constant azimuth angle ( about 30°), which is independent of the transmission distance.

# Supplementary Note 9: Comparison of different AV communication techniques in respect of the capacity and OAM range

**Table S3. Performance comparison of OAM communication techniques**

|  | Our work | Li [7] | Zhang [33] | Jiang [34] | Stank [35] | Wu [2] | Shi [23] |
| --- | --- | --- | --- | --- | --- | --- | --- |
| Active  / passive | Active, 16-elements array | Active, 16-elements array | Active, 20-elements array | Passive, 2-  passive structure | Active, 7-elements array | Active, 10-elements array | Active, 64-elements array |
| Principle | Phase-dislocation, machine learning | Orthogo-nality | Orthogo-nality | Reduce OAM order | Machine learning | Material de-modulation | Orthogonality |
| Multiplexed | Yes | Yes | Yes | No | Yes | Yes | Yes |
| Max OAM | 2.4 | 4.0 | 8.0 | 2.0 | 6.0 | 2.0 | 4.0 |
| Real time | Yes | Yes | Yes | Yes | No | Good | Yes |
| OAM interval | 0.2 | 1 | 1 | 1 | 1 | 1 | 1 |
| Capacity | 10-bit | 8-bit | 8-bit | 1-bit | 6-bit | 2-bit | 8-bit |
| OAM scope | ±0.6~±2.4 | -4.0~+4.0 | 1.0~8.0 | 1.0~2.0 | -3.0~+3.0 | -2.0~+2.0 | -4.0~+4.0 |
| OAM efficiency | 2.78 | 1 | 1.143 | 1 | 1 | 0.5 | 1 |
| Vortex-radius (λ) | 4.0 | 6.2 | 11.9 | 3.5 | 7.0 | 3.5 | 6.2 |
| Receiver number | 32 | 16 | 64 | 1 | 7 | 4 | 34 |

# Supplementary Note 10: Curves of accuracy and loss with various sampling points and FOAM resolutions


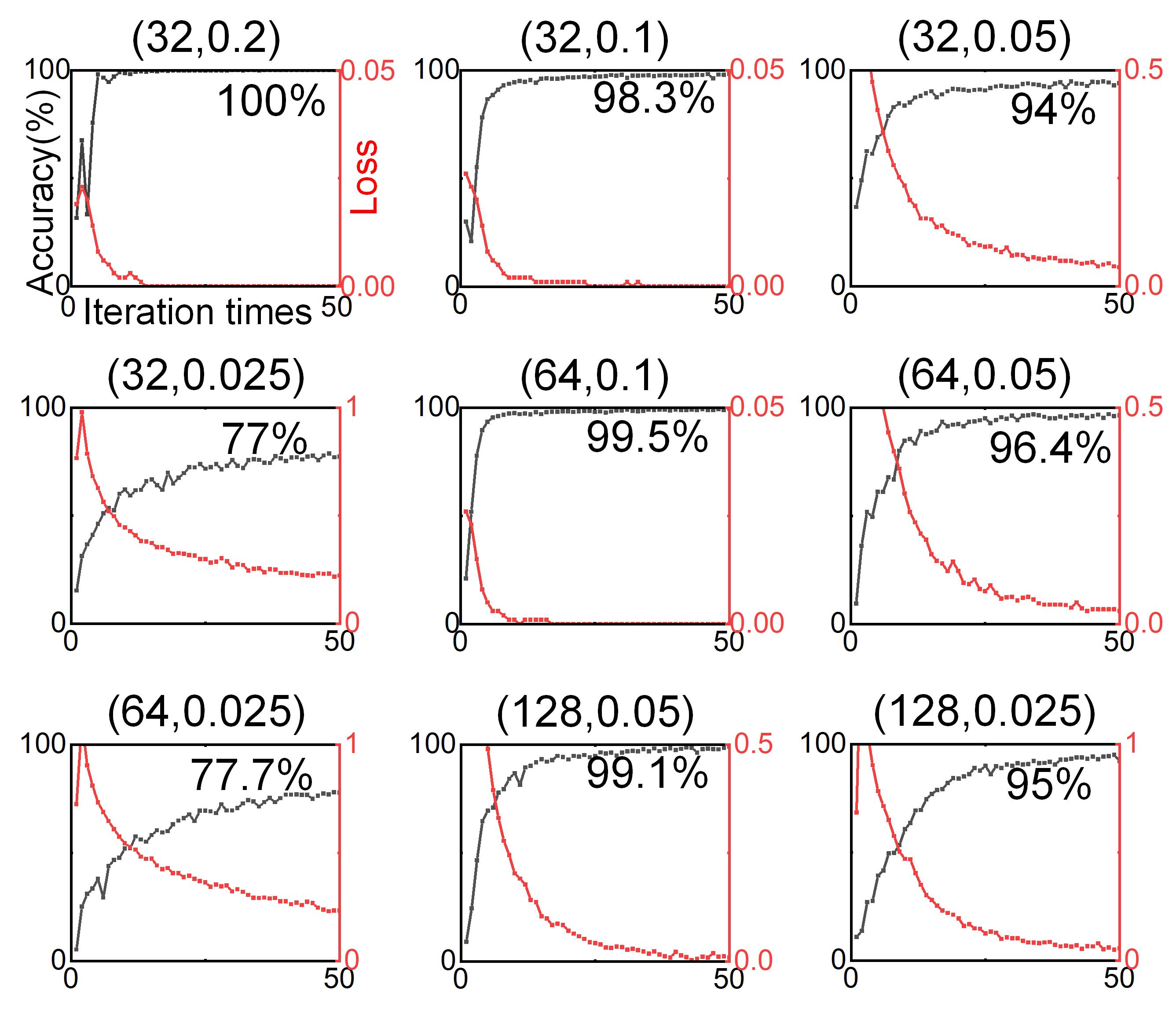


**Fig. S7. Curves of accuracy and loss with various sampling points and FOAM resolutions**. These curves are achieved with the FOAM resoltions of 0.2, 0.1, 0.05, and 0.025) using the single-ring sampling with the receiver numbers of 32, 64, and 128 for *l* = ±1.0 ~ ±2.0, which is corresponding to Fig. 2C.

# Supplementary Note 11: Architecture of CNN


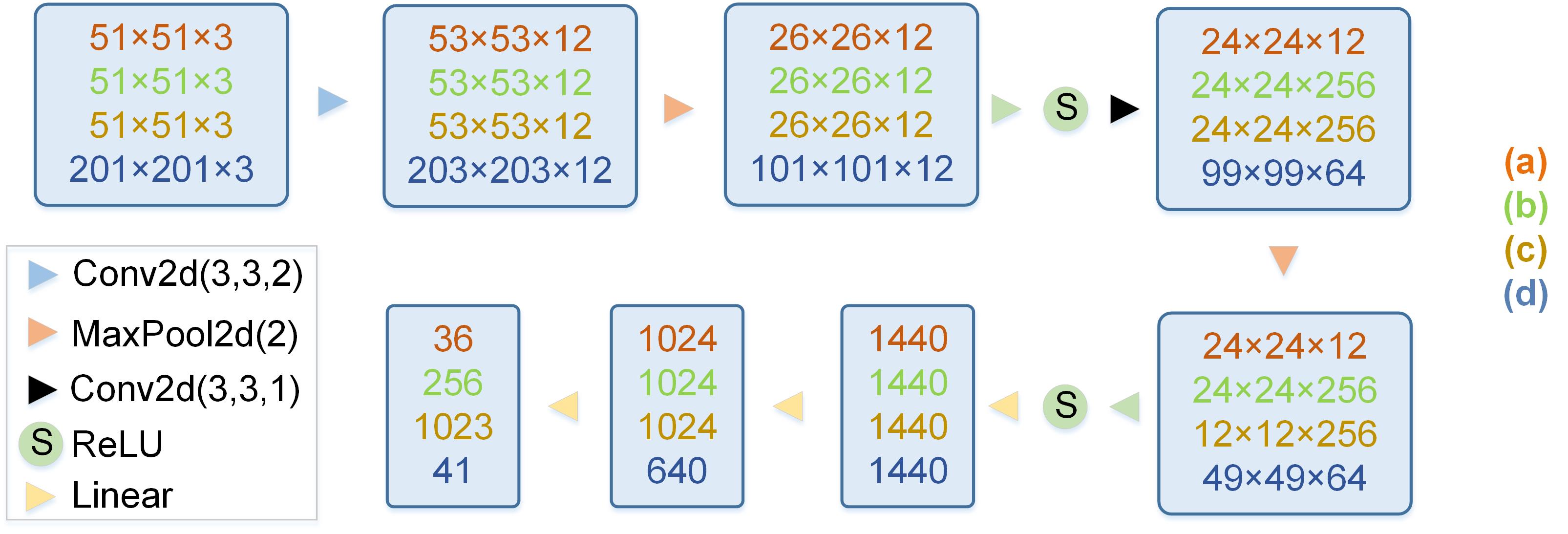


**Fig. S8. Network architecture used in this work.** The network architecture is used in FOAM decoding with the resolution of 0.2 and the channel capacity of **(a)** 5.17-bit for *l* = ± 0.8 to ± 2.2, **(b)** 8-bit for *l* = ± 0.8 to ± 2.2, and **(c)** 10-bit for *l* = ± 0.6 to ± 2.4. The parameters for each layer and operation are shown in the graph. The 2D pressure distributions acquired by the dual-ring sparse sampling are taken as inputs and classes as outputs. **(d)** The 2D pressure distributions acquired by the 128-point single-ring sampling are taken as inputs and classes as outputs to realize the high-resolution recognition of FOAMs with the accuracy of 0.025 for *l* = ± 1 to ± 2.
